# Management of neglected urethral stone and Fournier's gangrene as its complication: A case report

**DOI:** 10.1016/j.ijscr.2024.110233

**Published:** 2024-09-02

**Authors:** Anastasia Pearl Angeli, Soetojo Wirjopranoto, Yufi Aulia Azmi, Antonius Galih Pranesdha Putra, Kevin Muliawan Soetanto

**Affiliations:** a Faculty of Medicine, Universitas Airlangga, Surabaya, Indonesia; b Department of Urology, Faculty of Medicine Universitas Airlangga – Universitas Airlangga Academic Hospital, Surabaya, Indonesia; c Department of Health Sciences, University of Groningen, University Medical Center Groningen, Groningen, the Netherlands; d Department of Immunology, Faculty of Medicine Siriraj Hospital, Mahidol University, Bangkok, Thailand

**Keywords:** Fournier's gangrene, Urethral stone, Morbidity, Urinary diversion, Case report

## Abstract

**Introduction and importance:**

Fournier's gangrene (FG) is a rare necrotizing fasciitis, and it's a urological emergency. Another disease that can cause FG is urethral stones. This case report is prepared to discuss the management of neglected urethral stones and Fournier's Gangrene, as well as its complications.

**Case presentation:**

A 49-year-old male presented to the emergency room (ER) referred from the public health centre with a swollen and infected scrotum 2 weeks ago. It was worsened 1 day before hospital admission, accompanied by the discharge of pus from the scrotum. The patient also complained presence of intermittent fever, nausea, and vomiting. There was a history of straining when urinating. Physical examination showed a lump at the penis and crepitation at the scrotum. Radiological examination of the kidney ureter and bladder (KUB) x-ray and urethrography showed the presence of gangrenous gas at the scrotum. In this case, we perform open cystostomy, debridement necrotomy, and removal of urethral stone.

**Clinical discussion:**

Management of neglected urethral stones and Fournier's Gangrene cases needs to be done immediately to prevent poor outcomes. Necrotomy debridement management is performed immediately as a source of infection. Open cystostomy as a urinary diversion is performed so that urine does not pass through the urethra and the healing process of the urethra can be maximized.

**Conclusion:**

Controlling the source of infection and urinary diversion is important in cases where neglected urethral stones and Fournier's gangrene are found.

## Highlights

• Fournier’s gangrene on the penile region is rare
• Empirical antibiotics should be given to avoid mortality
• Simultaneous proscedures are needed for complex cases of penile FG followed by urethral stone.

## 1 Introduction

Fournier's gangrene (FG) is a relatively rare necrotizing fasciitis with a case count of 0.02 % of all hospital admissions. It is a rapidly progressive disease. FG causes problems in the deep and superficial tissues of the perineum, anal, scrotal, and genital areas. It has a strong predilection for men over women, with a ratio of 10 to 1, and is most commonly seen in men aged 50 to 79 [1]. FG is a fatal urological emergency that poses a serious risk to life [2]. Case data indicate that patients with Fournier's gangrene have a death rate of 20–40 %, with some series reaching 88 % [3].

The most frequent cause of Fournier's Gangrene is an infection of the subcutaneous tissue by polymicrobial organisms, which results in inflammation and, eventually, necrosis [4]. Other diseases can cause FG. Several risk factors are associated with the occurrence of FG, such as diabetes, alcohol abuse, immunosuppression, chemotherapy, chronic corticosteroid abuse, HIV, leukemia, liver disease, or debilitating diseases [5]. Urogenital stricture, indwelling catheter, traumatized catheterization, urethral stones, and prostate biopsy are among the causes of urogenital Fournier's gangrene. Without the proper tests, such as cystoscopy and urinary tract imaging, strictness and stones may not be detected or create noticeable symptoms [6]. On the other hand, stones may result in uncommon but deadly side effects, including resuscitation-needed obstructive uropathy, multi-stage surgery, or Fournier's gangrene [7].

A strong index of suspicion is necessary for the rare diagnosis of Fournier's gangrene, and prompt diagnosis is essential to improving patient outcomes [8]. A high mortality rate of up to 90 % results from delaying treatment because of the emergence of septic shock and its related consequences [9]. In this case, FG was caused by urethral stones that the patient had suffered from for a long time and had not been treated optimally. This case report is prepared to discuss the management of neglected urethral stones and Fournier's Gangrene, as well as its complications. This case report follows the SCARE Guideline 2023 [10].

## 2 Case presentation

A 49-year-old male presented to the emergency room (ER) referred from the public health centre with a swollen and infected scrotum 2 weeks ago. It was worsened 1 day before hospital admission, accompanied by the discharge of pus from the scrotum. The patient also complained presence of intermittent fever since 3 days ago, nausea, and vomiting. There was a history of straining when urinating and urine leakage from the scrotum since 6 months ago. The patient was diagnosed with LUTS 3 months ago, 6 months later the symptoms worsened and a lump was felt in the penis area. The patient suffered from uncontrolled diabetes mellitus type 2.

**Fig. 1:**
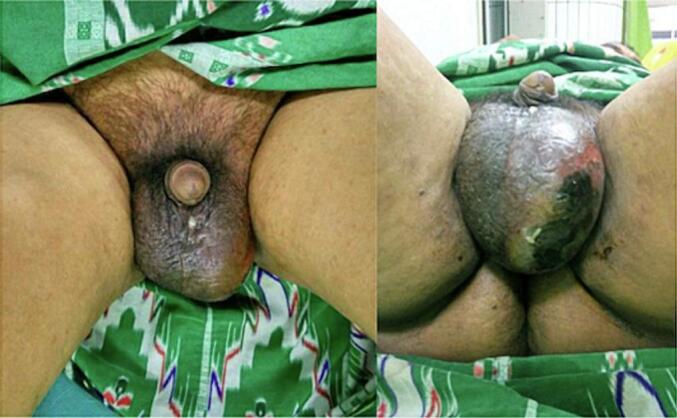
Clinical picture of the patient.

**Fig. 2:**
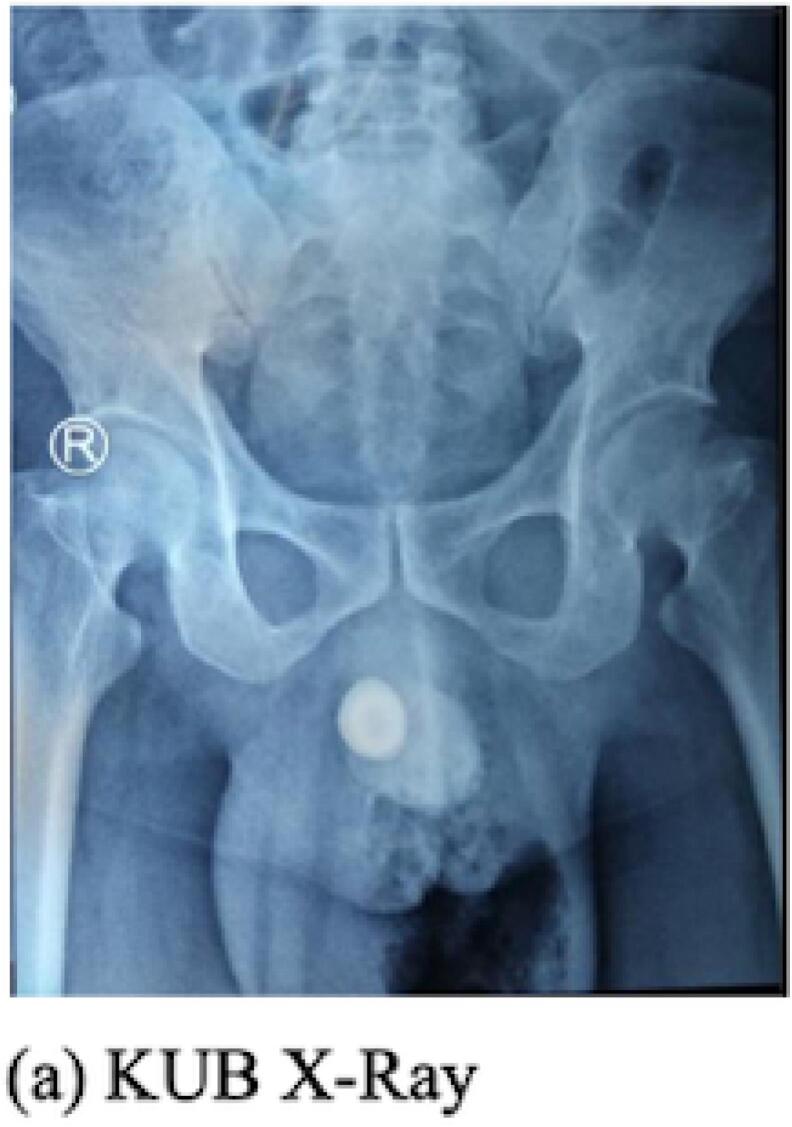

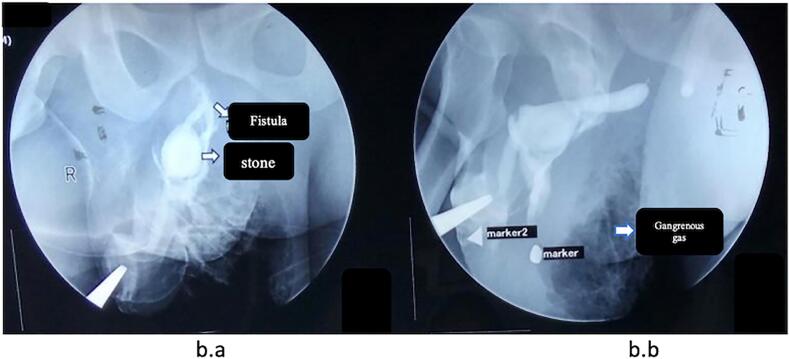
a. Kidney ureter bladder X-ray. b.a. Urethrography in the frontal position. b.b Urethrography in the lateral position.

Physical examination showed the patient's consciousness; there was a palpable lump at the penis and crepitation at the scrotum, also discharged pus from the scrotum (Fig. 1). Body temperature was 38.5°. Laboratory examination showed leucocytosis 37.5 × 10^3^ μL, creatinine serum 1.28 mg/dL. Laboratory results also showed Hba1C 9.5 %. Radiological examination of the kidney ureter and bladder X-ray (KUB) showed a urethral stone 2 × 3 cm (Fig. 2a), and urethrography showed the presence of gangrenous gas at the scrotum, urethral stone, and fistula (Fig. 2b). The size of the urethrocutaneous fistula is 0.5 cm on the ventral side of the urethra.

**Fig. 3:**
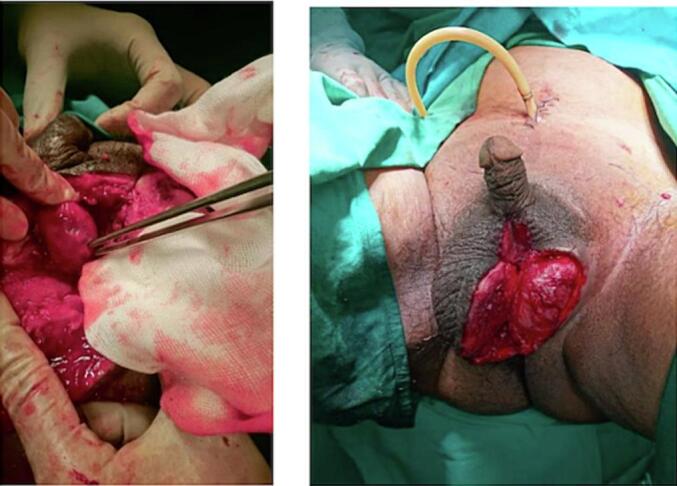
Debridement necrotomy and stone removal procedure.

In this case, we performed open cystostomy, debridement necrotomy, and removal of urethral stone (Fig. 3). To avoid mortality and worsening of the condition, the patient was given empirical antibiotics Ceftriaxone 1 g/12 h intravenous (i.v) and Metronidazole 500 mg/8 h intravenous (i.v). Open cystostomy started from the infra umbilical incision, the bladder was found, then a 20 fr catheter was installed. Clear urine production was 1500 cc/24 h. Continued with debridement of scrotal necrotomy, necrotomy was performed up to 1 cm beyond the edge of the scrotal skin with crepitation; pus was obtained +/− 200 cc. Necrotomy debridement was not towards the penis, only limited to the scrotum, 1 cm beyond the edge of the necrotic area, and did not explore the corpus cavernosum. Then, continued with penile exploration by performing a urethrotomy to remove the urethral stone.

Urethral stones were found, and they could be removed intact. The wound was sutured with interrupted 3.0 vicryl, and a 20 fr silicone catheter was installed. The use of polyglactin 910 (vicryl) in urethral sutures is done because the suture material is absorbable and does not need to be sutured, its tensile strength is good, the thread surface is smooth, minimizes trauma to the tissue, and there is no colonization of germs.

In patients, secondary stitches were performed on the wound from debridement, carried out on the 10th day after debridement necrotomy, without performing a skin graft. Postoperative and periodic wound care were carried out daily, using 0.9 % NaCl. Empirical antibiotics were given for 3 days; pus culture results showed *Escherichia coli*. Antibiotic therapy remained because it was sensitive to ceftriaxone and metronidazole. The duration of antibiotic administration was 7 days. Periodic laboratory evaluation was carried out on the 4th day; the results of leukocytes decreased to 21 × 10^3^ μL, and laboratory evaluation on the 7th day, leukocytes decreased to 9.2 × 10^3^ μL. Urethral catheter and cystostomy were maintained for 4 weeks. In the follow-up, the patient refused operative procedures such as cystourethroscopy or urethrography so only uroflowmetry was performed for evaluation during the check-up at the polyclinic.

## 3 Discussion

In this patient, an open cystostomy was performed for urinary diversion, during Durante debridement necrotomy, exposed stones were seen from the urethra, so a urethrotomy was performed to remove the urethral stones. The patient was given a urethral catheter so that it could enter the bladder. So for diversion through cystostomy and urethral catheter. Management of neglected urethral stones and Fournier's Gangrene cases needs to be done immediately to prevent poor outcomes. Necrotomy debridement management is performed immediately as a source of infection. Necrotomy debridement is intended to take pus specimens. This is to prevent infection [11]. Antibiotics and early surgical debridement of necrotic tissue are essential for FG treatment [12]. Open cystostomy as a urinary diversion is performed so that urine does not pass through the urethra and the healing process of the urethra can be maximized. Urinary diversion, either temporary or permanent, is necessary when urethral involvement is present [13].

To prevent urinary tract stones, patients are advised to: maintain a diet, if there is obstruction in the lower urinary tract then the obstruction must be treated, check if there are complaints of LUTS. Urinary tract stone illness can be prevented by first considering its genesis and risk factors. For all kinds of stones, low urine output and dehydration are common hazards; for calcium stones, the main dangers are hypercalciuria, hyperoxaluria, and hypocitraturia. Fluid intake (2.5–3.0 L/day), diuresis (>2.0–2.5 L/day), lifestyle and habit modification, and dietary management—such as limiting sodium at 2 or 3–5 g/day sodium chloride (NaCl), limiting oxalate-rich foods, avoiding vitamin C and vitamin D supplements, and limiting animal protein while increasing vegetable protein in patients—all play significant roles [14].

In the previous case, a 40-year-old man with lower urinary tract symptoms (LUTS) who got repeated primary healthcare visits and herbal treatments for ten years eventually acquired sepsis as a result of obstructive uropathy and Fournier's gangrene. The features of urethral stones, such as their size, location, and the existence of further urethral disease, determine how they should be treated. For several reasons, including the unavailability of lithotripsy equipment, the necessity to prevent urethral injury and bacterial spread, and the need for additional debridement and flap closure, the patient, in this case, underwent open surgery (urethrolithotomy) [7]. Another case found a 73-year-old guy who had a neglected urethral stone. Bilateral hydronephrosis, significant bladder distention, a stone in the mid-penile urethra, and air density in the abdominal wall's subcutaneous fat layer—all of which are compatible with necrotizing fasciitis—were confirmed by computed tomography. The infection was treated with parenteral antibiotics and sequential debridement in addition to immediate surgical debridement and cystourethroscopy. After the urethral defect was repaired due to necrosis, the skin defect was repaired, and the cleared area was rebuilt using a fasciocutaneous flap [15].

In this case, a silicone catheter was inserted. Bladder catheterization is actually performed for diagnostic and therapeutic reasons [16]. To prevent problems related to the procedure, the correct size, type, design, and training of the medical personnel are very important [17]. In this case, the catheter was used for 4 weeks. The previous research found that the length of time spent utilizing silicone urinary catheterization Silicone catheters can be left in place for up to eight weeks before replacement rather than the customary three-weekly change because Foley's catheters did not affect the rates of complications or symptoms [18]. There are, however, some issues to be mindful of. Significant morbidity, longer hospital stays, and higher medical expenses can result from indwelling catheters. While traumatic insertion and infection are frequent side effects, improvements in catheter design have reduced their frequency [19].

The patient has a risk of urethral stricture. This is because the patient has a chronic infection in this case, the patient also has a history of pus discharge from the urethra but has never been treated. Complaints of pus discharge from the urethra have been going on for 5 years, due to promiscuous sex habits. Urethral stricture has a variety of causes around the world, however, 15 % of cases are caused by inflammation and 19 % are related to trauma. Infection accounts for the majority of urethral stricture cases (66.5 %) in Nigeria, but only 15.2 % of cases in Brazil. Infection-related inflammatory urethral strictures often exclusively affect the anterior urethra and do not typically result in posterior urethral strictures. UTIs, among which *Escherichia coli* is the most often isolated pathogen, can also result in urethral strictures. In this instance, the same kind of bacteria was discovered [20]. According to other research, individuals with comorbid conditions like diabetes and hypertension should be advised that further curative measures would be required and that the chance of recurrence should be taken into account [21].

The use of polyglactin 910 (vicryl) in urethral sutures is done because the suture material is absorbable and does not need to be sutured, its tensile strength is good, the thread surface is smooth, minimizes trauma to the tissue, and there is no colonization of germs. The use of polyglactin 910 (vicryl) in urethral sutures is done because the suture material is absorbable and does not need to be sutured, its tensile strength is good, the thread surface is smooth, minimizes trauma to the tissue, and there is no colonization of germs. Polyglactin 910 is a commonly used absorbable braided multifilament suture that has better handling characteristics and is stronger than surgical sutures [22]. Polyglactin has better ligation and knotting ability in terms of soft tissue grafts [23].

Antibiotics are given empirically according to the hospital's germ map but then changed to culture-based. If the culture results are out, administration is carried out for 7 days according to the hospital's antibiotic guidelines. In this case, *Escherichia coli* was present, so ceftriaxone and metronidazole were given. The study found that the most susceptible bacteria were *Escherichia coli*, *Pseudomonas aeruginosa*, *Enterococcus faecium*, and *Staphylococcus aureus*. In addition, FG is a combination of mixed bacterial infections, which may be associated with rapid disease progression and severity. Broad-spectrum antibiotics should be selected as the basic antibacterial therapy. Generally, double or triple combinations are recommended as the basic criteria [24].

This case report has strengths and limitations. This case report can explain the management of urethral stones complicated by urethrocutaneous fistula accompanied by Fournier's gangrene. In this case report, there is a limitation, namely the limited generalization of the validity of the research and the impossibility of establishing a causal relationship related to the management of urethral stones with complications of urethrocutaneous fistula accompanied by Fournier's gangrene.

## 4 Conclusion

Management of neglected urethral stones and Fournier's Gangrene cases needs to be done immediately to prevent poor outcomes. Debridement necrotomy management was performed immediately as a source of infection. Open cystostomy as a urinary diversion is performed so that urine does not pass through the urethra and the healing process of the urethra can be maximized. Controlling the source of infection and urinary diversion is important in cases where neglected urethral stones and Fournier's gangrene are found.

## Ethical approval

Ethical approval has been acquired in this study by Health Research Ethics Committee of Dr. Soetomo General-Academic Hospital, Surabaya, Indonesia.

## Funding

This research did not receive any specific grant from funding agencies in the public, commercial, or not-for-profit sectors.

## Guarantor

Soetojo Wirjopranoto.

## CRediT authorship contribution statement

A.P.A.: Conceptualization, Methodology, Data curation, Investigation, Writing-Original draft preparation
A.G.P.P.: Conceptualization, Data curation, Writing-Original draft preparation
Y.A.A.: Data curation, Writing original draft-Reviewing, and Editing
K.M.S.: Data curation, Writing original draft-Reviewing, and Editing
S.W.: Writing original draft, Reviewing, Supervision, Validation.

## Declaration of competing interest

None declared.
